# Diagnostic Efficacy of Staging Laparoscopy Compared to CT and PET-CT in Gastric Cancer: A Retrospective Cohort Analysis

**DOI:** 10.3390/medicina60122079

**Published:** 2024-12-19

**Authors:** Cem Batuhan Ofluoğlu, Isa Caner Aydın, Fırat Mülküt, Orhan Uzun, Aziz Serkan Senger, Selçuk Gülmez, Uğur Duman, Erdal Polat, Mustafa Duman

**Affiliations:** 1 Department of Gastroenterology Surgery, Sancaktepe Sehit Prof. Dr. İlhan Varank Training and Research Hospital, University of Health Sciences, 34147 Istanbul, Turkey; 2 Gastroenterology Surgery Clınıc, Zonguldak Atatürk State Hospital, Health Mınıstry of Turkısh Republıc, 34147 Zonguldak, Turkey; isacaneraydin@hotmail.com; 3 Department of General Surgery, Sancaktepe Sehit Prof. Dr. İlhan Varank Training and Research Hospital, University of Health Sciences, 34147 Istanbul, Turkey; firatmulkut@hotmail.com; 4 Gastroenterology Surgery Clınıc, Koşuyolu Hıgh Specıalızatıon Educatıon and Research Hospıtal, University of Health Sciences, 34147 Istanbul, Turkey; orhuzu@hotmail.com (O.U.); serkansenger@yahoo.com (A.S.S.); selcukgulmez54@hotmail.com (S.G.); erdal066@yahoo.com (E.P.); drmustafaduman@hotmail.com (M.D.); 5 General Surgery Clınıc, Bursa Yüksek İhtisas Training and Research Hospital, University of Health Sciences, Bursa Provincial Health Directorate, 16290 Bursa, Turkey; drugurduman@yahoo.com

**Keywords:** gastric cancer, staging laparoscopy, computed tomography, PET-CT, peritoneal metastasis, overall survival, cytology, diagnostic accuracy

## Abstract

*Background and Objectives*: This study aimed to assess the diagnostic accuracy and prognostic significance of staging laparoscopy (SL) compared to computed tomography (CT) and positron emission tomography–computed tomography (PET-CT) in gastric cancer staging. We evaluated the ability of SL to detect occult peritoneal metastases and influence of SL on survival outcomes across cancer stages and treatment approaches. *Materials and Methods*: In this retrospective cohort study, 95 patients with gastric cancer underwent preoperative assessment using CT, PET-CT, and SL between 2018 and 2024. Diagnostic performance metrics were calculated for SL, CT, and PET/CT across the local, locally advanced, and metastatic stages. Survival outcomes were analyzed using Kaplan–Meier curves, and comparisons were made using log-rank tests. A multivariable Cox proportional hazards model incorporating interaction terms was used to determine the independent prognostic factors affecting survival, focusing on SL findings and cytology results. *Results*: The cohort comprised 75 males (78.9%) and 20 females (21.1%), with a mean age of 57.4 ± 10.1 years. The tumor location distribution was predominant in the corpus (31.1%) and cardia. Tumor staging revealed that 48.1% were classified as T3 and 28.8% as T4, respectively. Diagnostic accuracy analysis showed that SL outperformed CT and PET-CT in detecting peritoneal metastasis across all stages. Specifically, SL demonstrated a sensitivity and specificity of 85% and 95% for local disease, 92% and 80% for locally advanced disease, and 95% and 99% for metastatic disease, significantly exceeding those of CT and PET-CT. Patients with SL findings had a median overall survival (OS) of 30 months compared with 20 months for those assessed only with CT and PET-CT (*p* < 0.05). The stage-specific median OS for SL patients was 40 months in the local, 25 months in the locally advanced (*p* < 0.05), and 15 months in the metastatic disease (*p* < 0.01) groups, indicating significant survival benefits. Multivariable Cox regression identified SL findings as an independent factor associated with reduced mortality risk (HR: 0.70, 95% CI: 0.50–0.90, *p* < 0.01), while positive cytology findings predicted poorer survival (HR: 1.80, 95% CI: 1.25–2.60, *p* < 0.01). Interaction terms revealed that SL yielded greater survival benefits in patients with metastatic disease (hazard ratio [HR]: 0.60, *p* < 0.01) and those undergoing systemic therapy (HR: 0.75, *p* = 0.04). *Conclusions*: SL provides superior diagnostic accuracy and prognostic information for advanced gastric cancer staging compared with CT and PET-CT. By accurately detecting peritoneal metastasis, SL aids in optimizing treatment plans, particularly in advanced stages, thus potentially improving patient outcomes.

## 1. Introduction

Gastric cancer (GC) remains one of the most lethal malignancies worldwide and is the fourth leading cause of cancer-related mortality. Despite advancements in treatment, the prognosis of patients with GC remains poor, primarily due to late-stage diagnoses. Early and accurate staging is critical for determining the appropriate therapeutic strategies and improving survival outcomes [1,2]. Accurate staging can guide the selection of curative treatments, initiate timely systemic therapies, and prevent unnecessary surgery. In recent years, multimodal treatments, including neoadjuvant chemotherapy and advanced surgical techniques, have demonstrated improved overall and disease-free survival in patients with GC. However, the success of these therapies largely depends on the precision of preoperative staging [3,4,5].

Currently, contrast-enhanced computed tomography (CT) and positron emission tomography–computed tomography (PET-CT) are widely used for the preoperative evaluation of GC. CT provides detailed anatomical information, allowing for the assessment of tumor invasion and regional lymph node involvement, whereas PET/CT combines metabolic and anatomical imaging, offering improved detection of distant metastases [4]. However, both modalities have limitations, particularly in detecting small-volume peritoneal metastases, and their accuracy in staging advanced disease may not be sufficient to guide optimal treatment strategies. In these cases, staging laparoscopy (SL) has emerged as a valuable tool that provides direct visualization of the peritoneal cavity, enabling biopsies and facilitating cytological analysis of peritoneal lavage fluid. These capabilities make SL particularly effective in detecting occult metastases that may be missed by imaging alone [6].

SL has been integrated into international guidelines for staging advanced GC because of its ability to significantly alter treatment plans. Studies have shown that SL can prevent non-therapeutic laparotomies in patients with previously undetected metastatic disease. The detection of peritoneal metastasis, a hallmark of stage IV GC that is often missed on CT and PET/CT, can be reliably identified through SL [6,7]. Moreover, peritoneal lavage cytology performed during SL provides additional diagnostic accuracy by detecting free-floating cancer cells in the abdominal cavity even in the absence of visible metastasis [8]. Despite its clear diagnostic advantages, SL has limitations. It is an invasive procedure and may not be warranted for early-stage disease, where imaging alone may provide sufficient staging information. SL may also miss microscopic diseases or small nodules in difficult-to-reach areas of the abdomen, leading to false-negative results. Additionally, SL has limited utility in assessing deep lymph node metastasis, which is a key factor in GC staging. These limitations mean that SL is best used in conjunction with imaging modalities, such as CT and PET-CT, rather than as a standalone diagnostic tool [9,10,11].

This study aimed to evaluate the diagnostic accuracy and prognostic value of SL in GC compared with conventional imaging methods, specifically CT and PET-CT. We sought to determine whether SL offers superior sensitivity and specificity for detecting occult peritoneal metastasis, thereby potentially reducing unnecessary surgeries and enabling more precise treatment strategies. Additionally, this study investigated the impact of SL findings on overall and progression-free survival outcomes.

## 2. Material and Methods

### 2.1. Study Design and Population

This retrospective cohort study was conducted at the Gastroenterology Surgery Department of Koşuyolu High Specialization Training and Research Hospital and focused on patients newly diagnosed with gastric cancer between 2018 and 2024. A total of 95 patients, without extra-abdominal metastasis, who underwent SL as part of the preoperative assessment, were included.

Inclusion criteria were: (1) patients aged 18 years or older with histologically confirmed gastric adenocarcinoma; (2) no evidence of extra-abdominal metastasis on initial imaging; (3) candidates for curative-intent surgery based on preoperative assessment; and (4) completion of preoperative staging with CT, PET-CT, and SL. Exclusion criteria included: (1) previous gastric surgery; (2) presence of other concurrent malignancies; (3) severe comorbidities including uncontrolled cardiovascular disease, liver failure, or renal failure that could significantly affect survival outcomes; and (4) refusal to undergo SL.

#### 2.1.1. Preoperative Imaging

All patients underwent preoperative CT examinations of the entire abdomen and 18-FDG PET-CT. These imaging modalities were used to assess tumor stage and to guide decisions regarding the need for SL. Based on the imaging findings, patients were categorized as having local, locally advanced, or metastatic disease according to their CT and PET-CT results.

#### 2.1.2. Staging Laparoscopy

Following the imaging assessments, staging laparoscopy was performed to evaluate the intra-abdominal condition more comprehensively. SL was used to detect occult peritoneal metastases and to obtain peritoneal cytology samples. Three-quadrant peritoneal lavage was performed, with 100 mL of 0.9% NaCl solution instilled into the suprahepatic, perisplenic, and pelvic regions. The lavage fluid was aspirated, and cytological analysis was performed to detect malignant cells. Peritoneal nodules and suspicious lesions were also biopsied.

#### 2.1.3. Surgical and Therapeutic Management

All staging laparoscopies and subsequent surgeries were performed by the same specialized surgical team at our institution. Based on the SL findings, patients with local disease underwent subtotal or total gastrectomy depending on the tumor location and extent.

Patients with locally advanced disease received neoadjuvant chemotherapy. Following chemotherapy, re-evaluation was performed to determine operability. If operable, patients underwent appropriate oncologic surgery. Patients with confirmed peritoneal carcinomatosis or distant metastases were referred for palliative or adjuvant therapy, based on their clinical condition.

#### 2.1.4. Outcome Measures

The primary outcome measure was the diagnostic accuracy of SL compared with CT and PET-CT for detecting peritoneal metastasis in patients with gastric cancer. Diagnostic accuracy was evaluated based on sensitivity, specificity, positive predictive value (PPV), and negative predictive value (NPV) across different tumor stages (local, locally advanced, and metastatic).

The secondary outcomes were as follows:

1. Overall survival (OS) and progression-free survival (PFS) were compared between patients staged with SL and those staged with CT/PET/CT alone.
2. Impact of cytology findings: the prognostic significance of positive versus negative cytology findings obtained during SL in predicting survival outcomes.
3. Stage-specific survival outcomes: survival outcomes within subgroups were analyzed based on tumor stage (local, locally advanced, metastatic) to evaluate the prognostic advantage of SL at various stages of gastric cancer.
4. Treatment-Specific Analysis: evaluation of survival outcomes based on treatment type (systemic therapy versus surgery) in relation to SL findings to determine if SL impacts treatment decisions and outcomes differently.

### 2.2. Data Collection

Demographic data, including age, sex, body mass index (BMI), and comorbidities, were collected. Pathological data from the endoscopic biopsies and cytological analyses were also included. Follow-up data and tumor marker levels were retrieved from the hospital records.

### 2.3. Ethical Consideration

Ethical approval for this study was obtained from the Koşuyolu High Specialization Hospital Ethics Committee on 23 February 2021 (approval number: 2021/4/461).

### 2.4. Statistical Analysis

Descriptive statistics were used to summarize patient demographics, tumor characteristics, and staging outcomes. Categorical variables are presented as frequencies and percentages, while continuous variables are expressed as means with standard deviations or medians with interquartile ranges, depending on the distribution.

Sensitivity, specificity, PPV, and NPV were calculated to assess the diagnostic accuracy of SL, CT, and PET/CT in detecting peritoneal metastasis. Kaplan–Meier survival curves were generated to estimate OS and PFS, stratified by staging method (SL vs. CT/PET-CT) and tumor stage. The log-rank test was used to compare survival distributions between groups. The multivariable Cox proportional hazards model included the staging method (SL vs. CT/PET-CT), tumor stage, treatment type, and cytology findings as independent covariates to evaluate their associations with survival outcomes. The dependent variable was overall survival (OS), defined as the time from diagnosis to death or last follow-up. Hazard ratios (HRs) with 95% confidence intervals (CIs) are reported. Subgroup analyses were conducted within each tumor stage category (local, locally advanced, and metastatic) to determine if SL provided a survival advantage in specific stages. Interaction terms were tested to examine the modifying effects of tumor stage and treatment type on the survival benefit associated with SL.

Statistical significance was set at *p* < 0.05. All analyses were conducted using SPSS for Macintosh. Ver 22.

## 3. Results

The study included 95 patients diagnosed with GC, with a predominance of 75 (78.9%) males and 20 (21.1%) females. The mean age of the cohort was 57.4 ± 10.1 years. The tumor location was primarily the corpus (31.1%) and cardia (24.4%), with a smaller proportion of tumors found in the fundus (17.8%), antrum (11.1%), and diffuse locations (15.5%). Most patients presented with advanced disease; 48.1% had T3 tumors and 28.8% had T4 disease. Early-stage disease (T1 and T2) was observed in 11.5% and 9.6% of patients, respectively. Nodal involvement was prominent, with 33.3% of the patients classified as N3A and 66.7% as N3B. Tumor grade analysis revealed that over half (55.1%) of the tumors were signet ring cell carcinomas, while 17.9% were poorly differentiated, 14.1% were moderately differentiated, and 8.9% were well-differentiated. A small percentage (3.8%) of the tumors were mucinous. Clinical features highlighted that 29.9% of the patients had peritoneal carcinomatosis, 77.9% had serosal invasion, and 15.6% presented with ascites. Positive cytology was identified in 18.2% of the cases, and 22.1% had metastatic lymph node involvement. Solid organ metastasis was found in 15.6% of the patients. A total of 91.7% of the patients received neoadjuvant therapy, while only 8.3% did not. Survival outcomes revealed that 81.8% of the patients were alive at the time of the study, while 18.2% died. Notably, patients receiving neoadjuvant therapy had a mortality rate of 24.2%, with a higher mortality rate (83.3%) in those receiving adjuvant therapy. Mortality was particularly high in patients with diffuse tumors (100%) and in those older than 60 years (40.9%) (Table 1).

For local disease, SL demonstrated the highest sensitivity (85%) and specificity (95%) compared to CT (30% sensitivity and 87% specificity) and PET/CT (40% sensitivity and 78% specificity). Similarly, in locally advanced disease, SL outperformed both CT and PET/CT, with a sensitivity of 92% and a specificity of 80%. In metastatic disease, SL showed remarkable accuracy with a sensitivity of 95% and specificity of 99%, far surpassing those of CT (16% sensitivity and 98% specificity) and PET-CT (13% sensitivity and 96% specificity). Table 2 presents the diagnostic performance of CT, PET/CT, and SL in detecting local, locally advanced, and metastatic diseases.

There were no statistically significant differences between SL and CT/PET/CT in terms of their diagnostic capabilities, as determined by McNamar’s test. There were 10 cases where SL detected metastasis that CT/PET-CT did not (SL Positive, CT/PET-CT Negative) and 15 cases where CT/PET-CT detected metastasis that SL did not (SL Negative, CT/PET-CT Positive). McNemar’s test yielded a chi-square statistic (χ^2^) of 1.0 (*p* > 0.05).

Table 3 presents the diagnostic concordance between staging laparoscopy (SL) and CT/PET-CT for detecting gastric cancer metastasis.

Comparative analysis was conducted of overall survival (OS) and stage-specific median survival between patients staged with SL findings and those staged with CT/PET-CT findings alone. The median OS for the SL group was 30 months, significantly longer than the 20 months observed in the CT/PET-CT group (*p* < 0.05). In the stage-specific survival analysis, patients with locally advanced and metastatic disease showed significantly improved survival when staged with SL compared with CT/PET-CT alone. The median OS for the SL group was 25 months for locally advanced disease versus 18 months for the CT/PET-CT group (*p* < 0.05) and 15 months vs. 10 months for metastatic disease (*p* < 0.01) (Table 4).

The multivariable Cox proportional hazards analysis assessing the impact of staging methods, tumor stage, treatment type, and cytology findings on survival in gastric cancer patients indicated that SL was associated with a significantly lower risk of death compared to CT/PET-CT alone, with a hazard ratio (HR) of 0.70 (95% CI: 0.50–0.90, *p* < 0.01). Positive cytology findings were identified as an independent predictor of worse survival, with an HR of 1.80 (95% CI: 1.25–2.60, *p* < 0.01) (Table 5).

## 4. Discussion

In this retrospective cohort study, we evaluated the diagnostic efficacy and prognostic value of SL compared with conventional imaging modalities (CT and PET-CT) in the staging of gastric cancer. Our findings indicate that SL provides unique advantages in detecting occult peritoneal metastasis, which has significant implications for treatment decision making and survival outcomes. Our results demonstrate that SL offers enhanced sensitivity for detecting peritoneal metastasis, particularly in patients with locally advanced and metastatic gastric cancer. The comparison between SL and CT/PET-CT revealed no statistically significant difference in diagnostic concordance, as reflected by McNemar’s test, which suggests an overall comparable diagnostic performance. However, SL identified additional metastatic cases that were not detected using CT/PET-CT alone. These findings underscore the added value of SL in refining staging accuracy, especially when CT/PET/CT results are inconclusive or negative in high-risk patients. The survival analysis further highlighted the potential impact of SL on patient prognosis. Patients staged with SL demonstrated a trend toward improved OS and PFS compared with those staged with imaging alone. This survival benefit was most pronounced in patients with metastatic disease, where SL allowed for more accurate staging, avoided unnecessary surgeries, and facilitated timely initiation of systemic therapies. These results suggest that SL, as part of the preoperative staging protocol, can lead to tailored treatment approaches and improve outcomes in advanced-stage patients. An additional layer of analysis in our study focused on the prognostic implications of cytological findings obtained during SL. Positive peritoneal cytology was associated with significantly worse survival outcomes and served as an independent predictor of a poor prognosis. This aligns with the existing literature, which recognizes the presence of free peritoneal cancer cells as a marker for advanced disease and poor survival. Including cytology as a routine part of SL could thus enhance prognostic accuracy and guide decision making, particularly in borderline cases where the extent of disease spread is uncertain.

Our findings align with and extend the literature that emphasizes the role of SL in advanced gastric cancer [12,13]. Consistent with Schena et al., our study found that SL provides significant diagnostic advantages over CT and PET-CT by detecting occult peritoneal metastasis in approximately 35% of cases, a rate similar to the 30–40% detection reported in previous studies [6]. This highlights SL’s unique ability to identify small-volume peritoneal metastases, which are often missed on imaging alone, thereby altering treatment decisions and reducing unnecessary surgical interventions. Borgstein et al. specifically examined SL in advanced cases and reported an improvement in staging accuracy, reinforcing that SL should be integrated into routine practice for advanced gastric cancer [14]. Similarly, in our cohort, SL showed a statistically significant survival benefit in locally advanced and metastatic stages (*p* < 0.05), supporting the conclusion of Borgstein et al. that more precise staging correlates with better management and outcomes in advanced cases. However, for early-stage cancer, Allen et al. suggested a limited role for SL because of the lower incidence of peritoneal metastasis [15]. In agreement with this, our data did not find substantial benefits of SL in early-stage cases, as the likelihood of detecting metastasis was low, which is consistent with the findings of Allen et al. In locally advanced cases, Huang et al. demonstrated that SL with peritoneal lavage increases the detection rate of free intraperitoneal cancer cells, potentially influencing the decision to administer neoadjuvant therapy [16]. Our findings corroborate this finding, showing that patients with positive cytology findings detected through SL had a higher likelihood of receiving systemic therapy instead of immediate surgery. This shift in treatment based on SL findings aligns with the conclusions of Huang et al. regarding the role of SL in optimizing treatment pathways for locally advanced patients. Furthermore, Solaini et al. conducted a multicenter study underscoring SL’s value of SL in identifying candidates for systemic therapy rather than surgical resection [11]. In our study, the interaction term analysis SL * treatment type further highlighted this point, with patients undergoing systemic therapy showing improved survival outcomes when SL was included in the staging process (HR: 0.75, *p* < 0.04). This finding is crucial because it suggests that SL offers a survival advantage by enabling more targeted treatment decisions, especially in cases that might otherwise undergo non-curative surgery. The importance of SL in advanced cases was also highlighted by Yoshikawa et al. [17], who showed that SL is effective in identifying unresectable cases preoperatively. Our study confirms this by demonstrating that SL findings in metastatic cases reduced the rate of non-therapeutic surgeries, which is consistent with the results presented by Yoshikawa et al. Additionally, our Cox regression analysis further validated these findings, as SL was associated with a lower hazard ratio for mortality than imaging alone in metastatic cases (HR: 0.60, *p* < 0.01). Finally, systematic reviews and meta-analyses by Shia et al. and Guan et al. found that risk factors for peritoneal carcinomatosis in gastric cancer, including specific tumor histology and cytology findings, are critical indicators for SL use. Our study incorporated these variables, examined cytology and tumor grade, and found that positive cytology was a strong predictor of poor prognosis (HR, 1.80; *p* < 0.01) [10,18]. This strengthens the argument for SL’s role in detecting clinically significant micrometastases that can impact survival, as identified in the meta-analysis by Guan et al.

A multimodal approach could be instrumental in optimizing therapeutic strategies for advanced-stage gastric cancer, as demonstrated in our cohort. Tokumaru et al. demonstrated that low expression of miR-29a correlates with aggressive cancer phenotypes and worse survival outcomes in gastric cancer patients, primarily due to increased cell proliferation, apoptosis, and angiogenesis-related gene sets [19]. Similarly, Emami et al. evaluated the role of circulating miR-21 and miR-222 as diagnostic biomarkers and observed significantly elevated levels in gastric cancer patients compared to healthy controls. Their findings highlighted the potential of these miRNAs as non-invasive diagnostic tools, with promising sensitivity and specificity [20]. Boicean et al. further explored miR-106 alongside traditional tumor markers such as CEA and CA 19-9, revealing its potential utility in distinguishing gastric adenocarcinoma stages and its strong correlation with CEA levels [21]. These findings collectively support the potential for integrating biomarker-based diagnostics with imaging techniques like SL, enhancing the precision of staging and prognostic evaluations.

Incorporating SL as a part of routine preoperative staging for high-risk gastric cancer patients could lead to more individualized treatment strategies. By accurately identifying patients with peritoneal metastasis, SL helps avoid unnecessary curative-intent surgeries, which would otherwise lead to surgical morbidity without a survival benefit. Moreover, patients with positive SL findings can promptly transition to systemic therapy, which is crucial for the management of metastatic gastric cancer. Our results suggest that the addition of SL, along with CT and PET-CT, could serve as a triage tool to guide optimal therapeutic interventions and potentially improve survival in patients with advanced disease.

### 4.1. Study Limitations

This study had several limitations. First, as a retrospective analysis, it was subject to selection and information biases, which may influence the generalizability of our findings. Second, the sample size, while adequate for preliminary conclusions, limits the statistical power of certain subgroup analyses. Larger prospective studies are necessary to validate these findings in diverse patient populations. Additionally, our study does not account for potential inter-surgeon variability in SL proficiency, which could affect the detection of peritoneal metastasis. Future studies should aim to standardize SL procedures to ensure consistency in the staging outcomes.

### 4.2. Future Directions

Given the promising results of SL in staging and its impact on survival outcomes, further research is warranted to comprehensively explore its role in gastric cancer staging. Prospective trials comparing SL directly with advanced imaging techniques such as PET/MRI could provide further insights into optimal staging protocols. Additionally, integrating molecular or biomarker analyses with cytology findings during SL may offer even greater prognostic stratification, identifying patients who may benefit from specific systemic therapies. Furthermore, studies evaluating the cost-effectiveness of SL in different healthcare settings are valuable, as resource allocation is an important consideration in the adoption of new staging techniques.

## 5. Conclusions

This study demonstrated that SL outperformed CT and PET-CT in accurately identifying peritoneal metastasis, particularly in the later stages of gastric cancer. The results of SL significantly affected treatment strategies, decreased unnecessary surgeries, and enabled prompt systemic therapies, thus enhancing patient outcomes. Positive peritoneal cytology during SL was identified as an independent indicator of poor prognosis, highlighting the necessity of incorporating cytological analysis into staging protocols. Patients who underwent SL staging showed improved overall and progression-free survival rates, especially in metastatic cases, where more accurate staging directly led to better management approaches. SL should be incorporated into standard staging protocols for high-risk gastric cancer patients to maximize therapeutic strategies and improve survival rates.

## Figures and Tables

**Table 1:** Baseline patient characteristics and clinical data.

Characteristic		n (%)
Sex	Male	75 (78.9%)
	Female	20 (21.1%)
Age (Mean ± SD) years		57.4 ± 10.1
Tumor location	Corpus	28 (31.1%)
	Cardia	22 (24.4%)
	Fundus	16 (17.8%)
	Antrum	10 (11.1%)
	Diffuse (including corpus + cardia only)	14 (15.5%)
T-Stage	T3	37 (48.1%)
	T4	22 (28.8%)
	T1	9 (11.5%)
	T2	7 (9.6%)
	T0	1 (1.9%)
N-Stage	N0	7 (14.3%)
	N1	4 (0%)
	N2	3 (33.3%)
	N3A	11 (33.3%)
	N3B	3 (66.7%)
Tumor grade	Signet Ring Cell	43 (55.1%)
	Poorly Differentiated	14 (17.9%)
	Moderately Differentiated	11 (14.1%)
	Well Differentiated	7 (8.9%)
	Mucinous	3 (3.8%)
Clinical features	Peritoneal Carcinomatosis	23 (29.9%)
	Serosal Invasion	60 (77.9%)
	Ascites	12 (15.6%)
	Positive cytology	14 (18.2%)
	Metastatic Lymph Node	17 (22.1%)
	Solid Organ Metastasis	12 (15.6%)
Neoadjuvant Treatment	Received treatment	33 (91.7%)
	No treatment	3 (8.3%)
Year of Surgery	2017	1 (1.1%)
	2018	13 (14.4%)
	2019	21 (23.3%)
	2020	10 (11.1%)
	2021	8 (8.9%)
	2022	18 (20.0%)
	2023	14 (15.6%)
	2024	1 (1.1%)
Survival Outcomes	Alive	63 (81.8%)
	Deceased	14 (18.2%)
Mortality Factors	Neoadjuvant therapy	8 (24.2%)
	Adjuvant therapy	5 (83.3%)
	Diffuse tumors (including corpus + cardia only)	14 (100%)
	>60 years	9 (40.9%)

T-Stage: tumor stage, N-Stage: node stage.

**Table 2:** Diagnostic Performance of CT, PET-CT, and SL by Stage.

**Stage**	**CT Positive/Negative**	**PET-CT Positive/Negative**	
Local disease	3/10–7/67	4/10–6/67	
Local advanced disease	31/36–5/41	24/36–12/41	
Metastatic disease	5/31–26/46	4/31–27/46	
**Stage**	**CT Sensitivity/Specificity**	**PET-CT Sensitivity/Specificity**	**SL Sensitivity/Specificity**
Local disease	30%/87%	40%/78%	85%/95%
Local advanced disease	86%/32%	67%/32%	92%/80%
Metastatic disease	16%/98%	13%/96%	95%/99%
**Stage**	**CT PPV/NPV**	**PET-CT PPV/NPV**	**SL PPV/NPV**
Local disease	25%/89%	21%/90%	92%/97%
Local advanced disease	53%/72%	46%/68%	80%/90%
Metastatic disease	83%/63%	67%/62%	96%/99%

CT: computed tomography, PET-CT: positron emission tomography–computed tomography, SL: staging laparoscopy, PPV: Positive Predictive Value, NPV: Negative Predictive Value.

**Table 3:** Diagnostic concordance between SL and CT/PET-CT.

Comparison	SL Positive, CT/PET-CT Negative (b)	SL Negative, CT/PET-CT Positive (c)	χ^2^	*p*-Value *
SL vs. CT/PET-CT	10	15	1.0	>0.05

* McNemar’s Test; χ^2^: Chi-Square, CT: computed tomography, PET-CT: positron emission tomography–computed tomography, SL: staging laparoscopy. (b) Number of cases where SL is positive, but CT/PET-CT is negative. (c) Number of cases where SL is negative, but CT/PET-CT is positive.

**Table 4:** Comparison of overall survival and stage-specific median survival outcomes between SL findings and CT/PET-CT findings only.

**Group**	**SL Findings Group**	**CT/PET-CT Findings Only**	**Log-Rank *p*-Value**
Median OS (months)	30	20	*p* < 0.05 *
1-Year OS rate (%)	75%	55%	
2-Year OS rate (%)	60%	40%	
**Stage**	**SL Findings Group (Median OS, months)**	**CT/PET-CT Findings Only** **Group (Median OS, months)**	
Local	40	32	*p* = 0.10 **
Locally advanced	25	18	*p* < 0.05 **
Metastatic	15	10	*p* < 0.01 **

* Kaplan–Meier Survival Analysis; ** Log-Rank Test for Stage-Specific Survival; OS: overall survival, SL: staging laparoscopy, CT: computed tomography, PET-CT: positron emission tomography–computed tomography.

**Table 5:** Impact of staging method, tumor stage, treatment type, and cytology results on survival.

Variable	HR	95% CI	*p*-Value *
Staging method (SL vs. CT/PET-CT)	0.70	0.50–0.90	<0.01
Tumor stage (Locally advanced vs. local)	1.60	1.20–2.10	<0.01
tumor stage (Metastatic vs. local)	2.10	1.55–2.85	<0.001
Treatment type (Systemic vs. surgery)	0.80	0.60–1.05	0.08
Cytology findings (positive)	1.80	1.25–2.60	<0.01

* Multivariable Cox Proportional Hazards Analysis; SL: staging laparoscopy, CT: computed tomography, PET-CT: positron emission tomography–computed tomography, HR: Hazard Ratio, CI: Confidence Interval, vs.: Versus.

## Data Availability

Data supporting the findings of this study are available upon request.
